# Sib-pair subgroup familial type 1 diabetes mellitus in children in the state of Qatar

**DOI:** 10.1371/journal.pone.0271182

**Published:** 2022-07-08

**Authors:** Houda Afyouni, Basma Haris, Najeeb Syed, Ikhlak Ahmed, Noor Hamed, Tasneem Abdel-Karim, Shayma Mohammed, Amel Khalifa, Maryam Al-Maadheed, Mahmoud Zyoud, Ahmed Elawwa, Fawziya Al-Khalaf, Goran Petrovski, Khalid Hussain

**Affiliations:** Division of Endocrinology, Department of Pediatrics, Sidra Medicine, Doha, Qatar; Government College University Faisalabad, Pakistan, PAKISTAN

## Abstract

**Background:**

Type 1 diabetes is the most common type of diabetes mellitus (DM) in children. It can be sporadic in onset or cluster in families, which comprises parent-offspring and sib-pair subgroups. The risk of developing DM in first-degree relatives of affected individuals is 8–15 fold higher. There is limited data about familial DM from the Gulf region. This study aims to describe the clinical, biochemical and genetic characteristics of sib-pair familial type 1 diabetes in Qatar.

**Methods:**

Every child with DM following up at Sidra Medicine was recruited. Data was collected regarding clinical features, family history, type 1 diabetes autoantibodies and whole genome sequencing was performed. Genetic analysis for MODY genes and HLA association analysis was conducted.

**Results:**

44 families with sib-pair familial diabetes were identified. Of these, 2 families had 4 affected siblings and 5 families had 3 affected siblings. The majority are of Qatari ethnicity and the most common autoantibody was GAD65. The most common age of onset in the proband was 5–9 years while it was 10–14 years in subsequent siblings. The occurrence of DKA & HbA1c levels were lower in the second affected sibling. No relevant MODY gene variants were found. HLA analysis found 15 variants in at least 50% of the subjects. Most common were HLA-F*01*01*01G, HLA- DPA1*01*03*01G, HLA- DRB3*02*02*01G, HLA- E*01*01*01G & DRB4*03*01N.

**Conclusions:**

The prevalence of sib-pair diabetes is 3.64%. The second affected siblings were older. MODY is unlikely and Class I and II HLA genes was present in sib-pair diabetes.

## Introduction

Type 1 diabetes mellitus is a multifactorial disease in which genetic and environmental factors interact and lead to autoimmune destruction of pancreatic beta-cells resulting in hyperglycemia [1]. Type 1 diabetes mellitus is the most common type of DM in children with newly diagnosed cases estimated to be 98,200 children under 15 years of age in the world annually [2]. The incidence of type 1 diabetes mellitus is highest in Finland and Sweden and is also increasing at an alarming pace in the MENA region [3]. Association of HLA alleles with susceptibility to type 1 diabetes mellitus, has been the subject of intense investigations during the past decades and has resulted in the description of HLA alleles—DRB1*04, DQA1:03:01 and DQB1:03:01 as a strong indicator of the disease [4].

Type 1 diabetes can be sporadic in onset or cluster in families (referred to as familial type 1 diabetes). Familial type 1 diabetes comprises parent-offspring and sib-pair subgroups. Familial aggregation of type 1 diabetes accounts for more than 20% of the cases when the extended family is taken into consideration [1]. The prevalence of type 1 diabetes in individuals with an affected sibling by the age of 20 is approximately 4% compared to 0.4% of that in the general population [5–7]. In other words, the risk of developing type 1 diabetes increases by 8–15 fold with first-degree relatives and by 2 folds with second-degree relatives [1]. Familial clustering highlights the importance of genetics in the pathophysiology of the disease, attributed to a higher prevalence of the HLA and non-HLA genes in affected family members. On the other hand, the low concordance rate in monozygotic twins suggests that other factors are involved in the pathogenesis of the disease [8].

The risk of developing familial type 1 diabetes is higher in the offspring of affected fathers and those of affected mothers [9–11]. Sporadic type 1 diabetes tends to have a more aggressive course at presentation as compared to familial type 1 diabetes [10, 12, 13]. In siblings, the risk of developing type 1 diabetes is higher with younger age of index at diagnosis [5, 14]. It has been suggested that the pathogenesis of type 1 diabetes differs in both sporadic and familial cases, but the data from different studies are inconsistent [1, 13, 15]. In Finland, 2 studies have shown that the type 1 diabetes antibody profile is similar between familial and sporadic cases of diabetes which suggest a similar pathogenetic process for beta cell destruction and that better metabolic decompensation at presentation for familial cases can be explained by the parental awareness of diabetes symptoms [13, 15]. Lebenthal et al [12] on the other hand even suggested that the pathophysiology between the 2 familial subgroups: sib-pair and parent-offsprings might be different.

There is limited data on familial forms of type 1 diabetes from the Gulf and Middle-East and North Africa (MENA) regions. A retrospective review of cases from Qatar found that the prevalence of sib-pair type 1 diabetes was 14.6% [16]. Another retrospective study conducted in Oman showed that a family history of type 1 diabetes was present in 22% of the cases [17]. Finally, a study from Kuwait reported a familial form of type 1 diabetes in 33% of their patients with type 1 diabetes [18].

In this prospective study, we identified all patients with sib-pair group type 1 diabetes attending a regional diabetes centre. This study aims to comprehensively describe the clinical, biochemical, immunological and genetic characteristics of these sib-pair groups of children and adolescents with type 1 diabetes.

## Material and methods

### Ethical compliance

This study was approved by the Institutional Review Board (IRB) for the protection of human subjects, approval number 1702007592. Written informed consent and assent were obtained as necessary from patients and family members.

### Selection and recruitment of participants

In this prospective study, every child with DM (aged 0–18 years) attending the diabetes clinics or admitted as an inpatient in Sidra Medicine, which is the only pediatric diabetes centre in Qatar, was recruited from 2018–2020. To confirm that all children with diabetes are captured, all previous hospital records were examined. Clinical details about the birth history, gestational age, ethnicity, age of onset of DM, family history, BMI, weight, signs of insulin resistance (acanthosis nigricans) and other system involvement were collected and documented. Information on the family history of all types of diabetes was obtained from the families by using a questionnaire.

**Fig 1:**
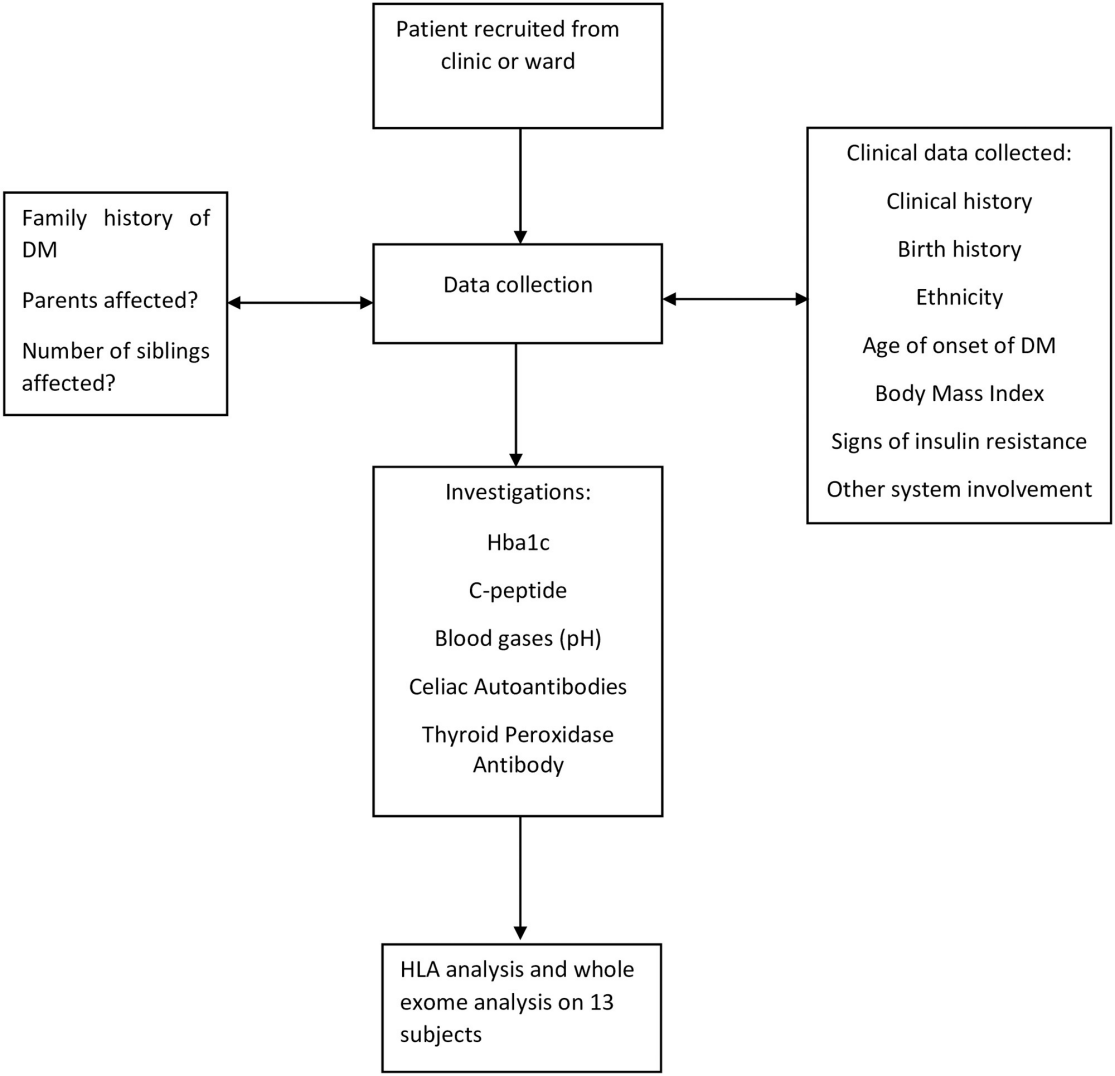
Flowchart describing the methodology.

Peripheral blood samples were collected for complete antibody profiling-all 4 autoantibodies namely Glutamic Acid Decarboxylase 65 (GAD65), Insulin Auto Antibody (IAA), Islet Antigen-2 Auto Antibody (IA-2A) and Zinc Transporter 8 (ZnT8A) were measured and titres recorded, C-peptide, celiac and Thyroid Peroxidase (TPO) antibodies are also measured. Blood samples were also collected for extraction and storage of serum, plasma, DNA and RNA for further studies. With the help of clinical history and antibody assays, based on ADA guidelines, the patients were classified as having type 1 diabetes [19]. A flowchart of the methodology is shown in Fig 1.

### Antibody assay methodology

Complete Antibody profiling of every child with diabetes in Qatar was performed and titres were recorded. This was done at the time of recruitment into the study for all known type 1 diabetes patients while newly diagnosed cases were tested at the time of diagnosis before starting insulin treatment.

GAD65-Radioimmunoassay was performed. (125)I-labeled recombinant human glutamic acid decarboxylase (GAD65) is incubated with the patient’s diluted serum. Antihuman IgG and IgM are then added to form an immunoprecipitate. After washing the precipitated immune complexes, specific antibodies are detected by counting gamma-emission from the pellet’s bound (125)I-GAD65 [20].

Insulin autoantibody-Radioimmunoassay performed. (125)I-labeled recombinant human insulin is added to the test serum; if the antibody is present, it forms a soluble complex with the labeled insulin. Subsequent addition of goat antihuman IgG and IgM precipitates the complex. The amount of radioactivity in the precipitate is proportional to the level of antibody in the serum.

IA-2 autoantibody- Radioimmunoassay performed. (125) I-labeled recombinant human IA-2 is added to the test serum; if the antibody is present, it forms a soluble complex with the (125) I-labeled IA-2. Subsequent addition of goat antihuman IgG and IgM precipitates the complex. The amount of radioactivity in the precipitate is proportional to the level of antibody in the serum [21].

Zinc Transporter 8 (ZnT8) autoantibody- Enzyme immunoassay. Zinc Transporter 8 (ZnT8) antibodies are principally directed against the C terminal domain of ZnT8. The ZnT8 autoantibody ELISA is based on the bridging principle that employs the ability of divalent ZnT8 autoantibodies to bind to ZnT8 coated onto the plate well with one arm, and to liquid ZnT8-biotin with the other arm. Calibrators or undiluted serum samples in duplicate are added to ZnT- coated plate wells and incubated overnight. ZnT8-biotin is added to each well and plate. After another incubation, aspiration, and wash, streptavidin-peroxidase is added to each well. Another incubation, aspiration, and wash are performed and peroxidase substrate is added. After a final incubation, 0.5 mol/L H2S04 stop solution is added to each well. Absorbance is measured at 450 nm, blanked against wells containing peroxidase substrate and H2S04 only.

### Genetic testing methodology

Peripheral blood specimen was collected and DNA samples were extracted from all individuals recruited into the study including patients and parents in Sidra Medicine. Whole Exome Sequencing was conducted on 13 subjects and sequenced on Illumina HiSeqX platform using a 150-base paired-end single-index-read format. Reads in FASTQ files were then mapped to the NCBI human reference genome GRGh37/hg19 using Burrows-Wheeler Aligner (BWA-MEM) version 0.7.8. All subjects underwent variant calling using GATK(v3.6) and annotation was performed using SNPEf [22]. Variants file was normalized and decomposed using vt [23]. Additionally, vcfanno [24] was used to annotate the VCF files with extensive available data resources like gnomad gnomad, exomes.r2.0.2, gnomad.genomes.r2.0.2.sites, 1K genome, Exac etc. Genomic variants belonging to 53 genes already known to be implicated in MODY were extracted for patient samples from the multisample VCF file. These variants were further filtered for non-exonic regions using exome bed (Exome-Agilent_V6) and only variants belonging to exome regions were retained for downstream analysis. We looked for the non-synonymous variants which are absent or present in a frequency less than 0.1% in public databases.

### Extraction of HLA data from WGS methodology

We used Population Reference Graph (HLA-PRG) [25] to identify HLA alleles from the whole genome sequencing of 13 samples. The accuracy of HLA genotypes was assessed on a family-based approach. The association of HLA alleles with type 1 diabetes phenotype in each group was tested using fisher’s exact test in R.

## Results

**Fig 2:**
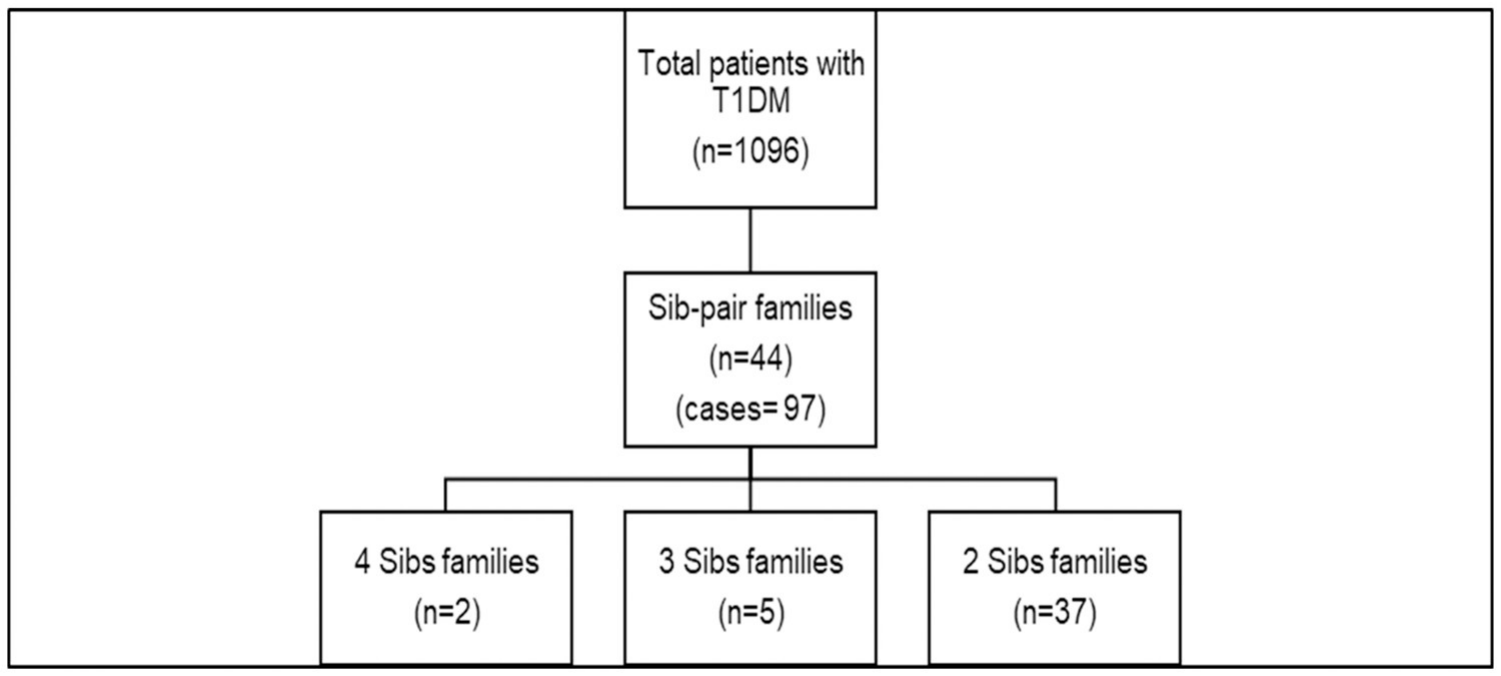
Representation of familial T1DM cases.

**Fig 3:**
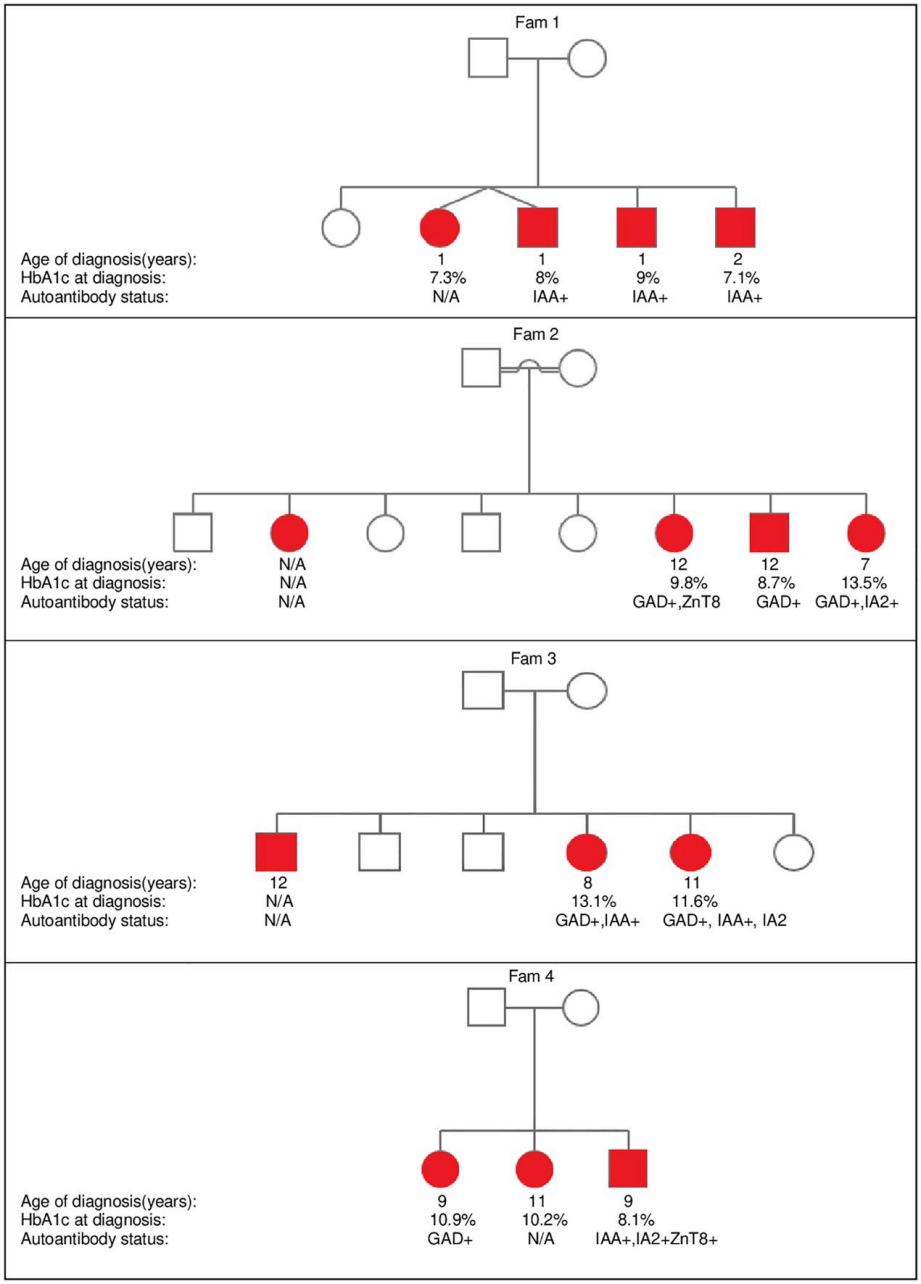
Pedigree illustration of families with more than 2 affected siblings.

The total number of children and adolescents (aged 0–18 years) with confirmed type 1 diabetes attending the Sidra diabetes service from 2018–2020 was 1096. In this cohort, we identified 44 families with a total of 97 patients with sib-pair autoantibody positive type 1 diabetes. This represents 9% of the total number of type 1 diabetes patients in our Center (Fig 2). Of these families, 7 had more than 2 affected children (Fig 3).

### Clinical characteristics of patients

**Fig 4:**
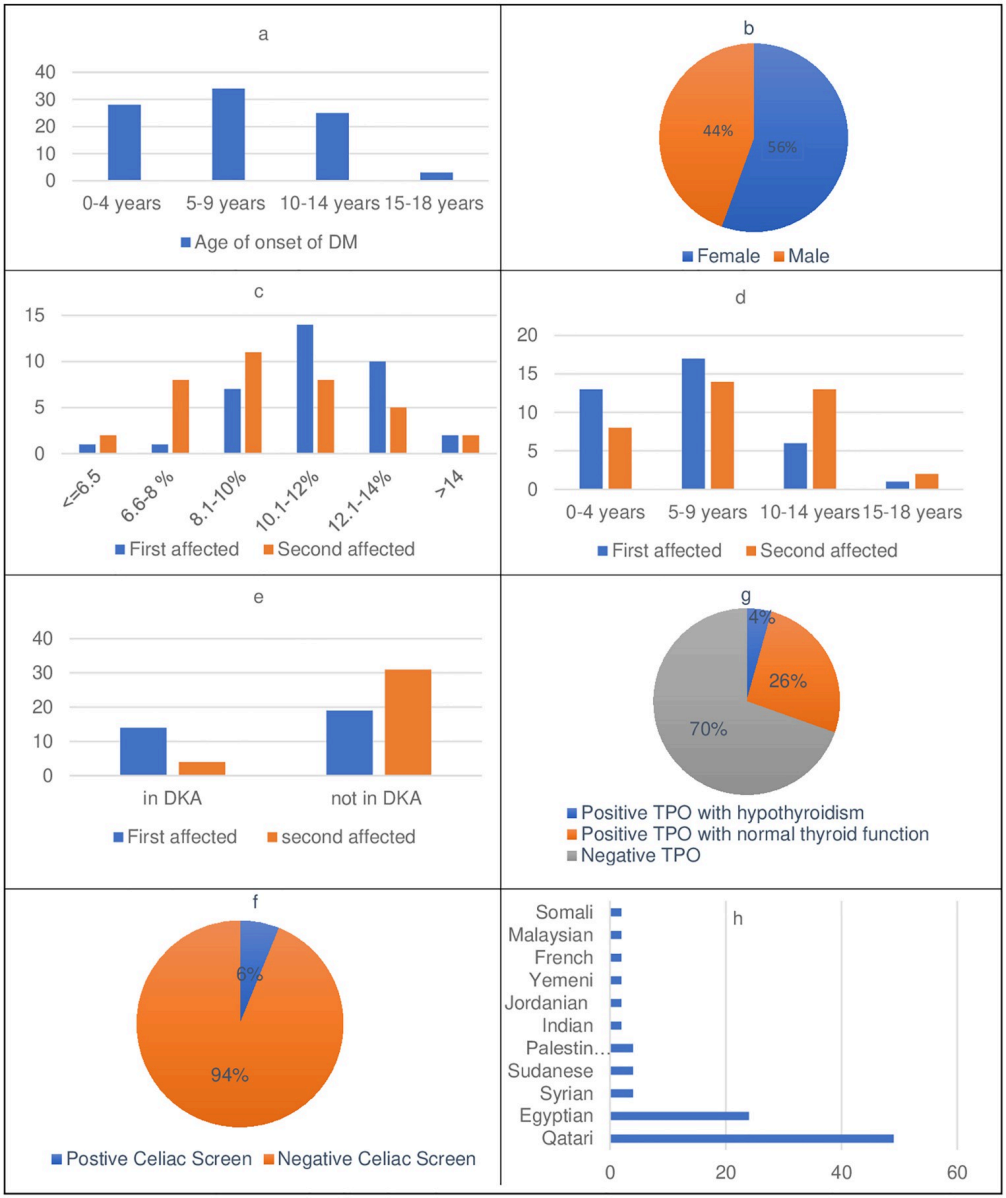
Clinical features observed in familial T1DM cohort. a Age of onset of DM. b Gender distribution. c HbA1c at presentation in first Vs second affected sibling. d Age of onset in first Vs second affected sibling. e DKA at presentation in first Vs second affected sibling. f Celiac screen in the cohort. g Thyroid function and TPO status in the cohort. h Ethnicity.

The clinical features of the patients along with a comparison of the proband and second-affected family members are summarized in Fig 4. The majority of our cohort were Qatari patients followed by Egyptian as the second most common nationality. Almost all the probands had onset of DM below the age of 14 with 5–9 years the most common age group followed by 0–4 years. However, the second-affected siblings were older at the time of diagnosis as their age of onset was mostly at 5–9 years followed by 10–14 years of age.

At presentation, 23% of patients had DKA and HBA1c ranged from 8–12%. The proband group had worse metabolic decompensation since 6 patients had moderate DKA with pH of 7.1–7.2 and 3 had severe DKA with pH of <7.1 while in subsequent siblings only one patient each had moderate and severe DKA. HBA1c levels were higher in probands (average 11.13%) than siblings (average 9.55%). More frequent DKA episodes were also observed in probands (n = 15) when compared to subsequent siblings (n = 4).

The patients with positive TPO represent 28% of the total cohort including probands and subsequent siblings (n = 28) but among those, only 4 patients had clinical hypothyroidism on treatment. Celiac screen with tissue transglutaminase antibodies (TTG) was positive in 6 patients in the whole cohort. Detailed TPO and TTG antibody status of families with >2 affected siblings is shown in Table 1. 8 patients had at least one affected parent while 2 had both parents with DM.

**Table 1:** TPO and Celiac autoantibody status in families with >2 affected siblings.

Patients	TPO status	Celiac
**Family 1**		
Sibling 1	Negative	Negative
Sibling 2	Negative	Negative
Sibling 3	Negative	Negative
Sibling 4	Negative	Negative
**Family 2**		
Sibling 1	N/A	Negative
Sibling 2	Positive	Negative
Sibling 3	Positive	Negative
Sibling 4	Negative	Negative
**Family 3**		
Sibling 1	N/A	Negative
Sibling 2	Positive	Negative
Sibling 3	Positive	Negative
**Family 4**		
Sibling 1	Positive	Negative
Sibling 2	Positive	Negative
Sibling 3	Negative	Negative
**Family 5**		
Sibling 1	N/A	N/A
Sibling 2	N/A	Negative
Sibling 3	Positive	Negative
**Family 6**		
Sibling 1	Positive	Negative
Sibling 2	Positive	N/A
Sibling 3	Negative	Positive
**Family 7**		
Sibling 1	Negative	Negative
Sibling 2	Positive	Negative
Sibling 3	Negative	Negative

### Autoantibody status

**Fig 5:**
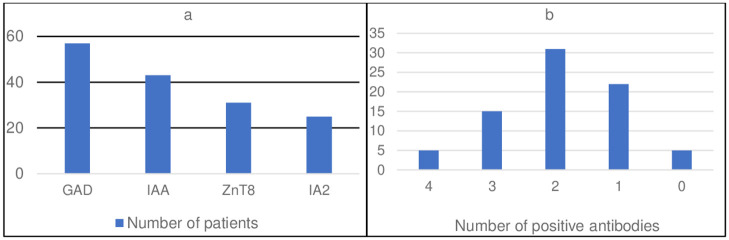
Autoantibody status of sib-pair familial DM. a Type of autoantibody present. b Number of autoantibodies present.

All 4 type 1 diabetes antibodies namely GAD65, IAA, IA2 and ZnT8 were done in 79 patients (81% of the total cohort). GAD65 was the most common antibody followed by IAA and ZnT8 (Fig 5).

### HLA haplotype analysis

Following alleles were found to be present in more than 50% of the patients where AF is the allele frequency for the allele in each patient (Table 2).

**Table 2:** HLA alleles found in the cohort.

HLA-Allele	AF	Allele Present (HOM/HET)
F*01*01*01G	100%	100%
DPA1*01*03*01G	96%	100%
DRB3*02*02*01G	88%	92%
E*01*01*01G	71%	100%
DQB1*02*01*01G	63%	92%
DRB4*03*01N	67%	100%
DPB1*04*01*01G	50%	75%
DQA1*05*01*01G	46%	92%
DRB1*03*01*01G	46%	92%
G*01*03*01G	46%	83%
DPB1*03*01*01G	38%	58%
DRB4*01*01*01G	33%	67%
DQA1*03*01*01G	29%	58%
E*01*03*01G	29%	58%
C*06*02*01G	29%	50%

### Genetic analysis

The affected members of all families were analysed as a whole cohort to look for common MODY genes (Table 3). Families were also analysed individually. No relevant MODY gene variants were found in fam 1, fam 2 or fam 3. Table 4 shows the variants present in fam 8 in a heterozygous state.

**Table 3:** MODY gene variants present in at least 10% of the cohort.

Gene	Variant	Allele Frequency in cohort
HNF1B	n.-1954G>C	0.25
HNF4A	n.506+2275_506+2276insGA	0.25
BLK	c.-2+5745G>A	0.16
BLK	c.-2+7277_-2+7278delGT	0.16
BLK	c.-2+7280_-2+7281insAA	0.16
ABCC8	c.822+339G>T	0.16
NEUROD1	c.*677A>T	0.16
APPL1	n.*2030C>T	0.16

**Table 4:** Variants of unknown significance present in fam 8.

Gene	Variant	Effect
HNF1A	c.1418G>A	variant of unknown significance
CEL	c.1558G>A	variant of unknown significance
CEL	c.1693C>T	variant of unknown significance

## Discussion

Familial clustering is a well-known fact among patients with type 1 diabetes. In siblings, the risk of developing type 1 diabetes by the age of 30 years is 5.5% and increases to 6.9% by the age of 50 years [5]. The risk is even higher according to a Danish study [26] where the risk is 6.4% by the age of 30 years and goes up to 9.6% by the age of 60 years. Patients with no family history of diabetes have a worse metabolic presentation when compared to familial cases [10, 12, 13] and children with affected fathers have worse presentation than those with affected mothers [10]. Harjutsalo et al also showed that other risks for siblings DM are male gender and older paternal age at delivery [5].

In Qatar, the high rate of consanguinity (54%) [27] provides a good opportunity to study familial diabetes as “theoretically” it will increase the aggregation of HLA and non-HLA genes related to diabetes among families. Only a few studies about familial diabetes have been conducted in the MENA region and to our knowledge, this is the first study that describes the sib-pair subgroup in familial diabetes. A retrospective review in Qatar showed that the prevelance of sib-pair type 1 diabetes was 14.6% with a male predominance and an earlier age of onset in familial vs nonfamilial groups with positive anti-islet antibody [16]. However, in this study, only two autoantibodies were measured (GAD65 and Islet) for the diagnosis of type 1 diabetes. In Oman, another retrospective study showed a positive family history of type 1 diabetes in 22% of the cases and that the initial presenting symptoms (such as polyuria, polydipsia, weight loss and diabetic ketoacidosis) did not differ between familial and non-familial cases [17]. In this study, the diagnosis of type 1 diabetes was not based on autoantibody profiling. In Saudi Arabia, a novel mutation was discovered in a Saudi family with 3 affected siblings [28] suggesting a possible genetic basis for familial type 1 diabetes. Finally, a study from Kuwait reported a familial form of type 1 diabetes in 33% of their patients with type 1 diabetes but again the diagnosis of type 1 diabetes was not based on autoantibody status [18].

We had 44 index cases who had at least one sibling with type 1 diabetes, which represents a prevalence of 3.64% of our diabetic cohort. This result is comparable to other studies in Finland and Denmark where the prevalence of affected siblings is approximately 5% [1, 29]. On the other hand, our result is much less than the prevalence of familial cases noticed in other gulf countries like Kuwait (15.8%) [18], Oman (22%) [17] and Saudi Arabia studies ranged from 11%–26.3% [30]. This can be explained by the fact that we analyzed only the sib-pair group while the latter studies included affected parents and siblings with/without second-degree relatives. In addition, these studies from the GCC did not measure diabetes autoantibodies in all affected parents and sib-pairs, so it is not clear if all the reported patients had autoantibody positive type 1 diabetes.

Most of our probands presented with diabetes were in the age group of 5–9 years while subsequent siblings were diagnosed most commonly in the 5–9 years and 10–14 years age group. The mean age at diagnosis in all affected ranged between 7.6–8.6 in previous studies [1, 9, 29] while Lebenthal et al showed older mean age at presentation of 9.7 years [12].

In concordance with previous studies, we found that the second affected siblings are older at diagnosis than the probands [12, 13, 26, 29]. Despite the consistent data, the reason behind this phenomenon still needs clarification. Harjutsalo et al [5] even stated that an early age at diagnosis of index cases is linked with an increased risk of developing type 1 diabetes in subsequent siblings.

In terms of gender, there is a slight female preponderance in our cohort (56%). While this result agrees with the data from Lebenthal et al [12], data from other studies have been inconsistent. Studies from Pittsburgh [13] and Finland [1, 9] showed no difference in gender distribution in familial nor sporadic cases. On the other hand, the Danish study [29] found a significant preponderance in males in sporadic cases while no difference in familial cases.

In general, previous studies have shown that patients with first or second-degree relatives with type 1 diabetes tend to have less metabolic decompensation at presentation than those without a positive family history of type 1 diabetes [1, 12, 15]. In our cohort, the overall percentage of DKA at presentation was 23%. This is comparable to Lebenthal et al [12] results of 23.8% among the sib-pair group. When compared, the proband cases had a worse presentation as 42% of them came with DKA Vs 11% in the second affected sibling. HBA1c at presentation was mostly between 10.1–12% in probands Vs 8.1–10% in their affected siblings. This result can be explained by the better awareness of caregivers about diabetes symptoms and the readily available glucose-measuring devices. However, the fact that 11% of the second affected siblings presented with DKA highlights the importance of parental education and awareness. Two recent studies by Lebenthal [12] and Turtinen [9] showed that IAA antibodies were more frequent in familial cases in comparison to sporadic cases. However, most of the previous studies showed no difference between sporadic and familial cases in terms of the presence of type 1 diabetes autoantibodies. The most frequent antibody encountered in our cohort was GAD65 antibodies and the least one was IA2 antibodies. Most of our patients had 2 positive antibodies.

Our genetic analysis found some variants in genes that cause MODY, however, they were all variants of uncertain significance or predicted to be benign and as such cannot be attributed to being disease-causing without further clinical and laboratory evidence. Also, we did not find any common genetic abnormality in any of the affected siblings in all the families in our cohort. Hence we think it is safe to say there is no need to screen for MODY if autoantibodies are positive on sib-pair cases.

HLA genes are reported to cause 40–50% of familial aggregation of type 1 diabetes in previous studies with the most common being polymorphisms in class II HLA genes which encodes for HLA-DQ and DR [31]. Our HLA genetic analysis found 15 variants found in at least 50% of the affected probands and siblings in the whole cohort, the most common being HLA-F*01*01*01G, HLA- DPA1*01*03*01G, HLA- DRB3*02*02*01G, HLA- E*01*01*01G & DRB4*03*01N. A limitation of our study is the relatively small number of subjects, hence the significance of this finding is uncertain.

## Conclusions

This is the first study from the state of Qatar to characterize the clinical, epidemiological, immunological and genetic aspects of familial sib-pair autoantibody positive type 1 diabetes in detail. The clinical and immunological findings in our patients correlate with previously published data on the sib-pair form of type 1 diabetes. In addition, our genetic findings show that MODY forms of diabetes are rare in sib-pair antibody positive type 1 diabetes.

### Future perspective

The age of onset of diabetes is later in second affected siblings and their diabetes is better controlled. Early age at diagnosis of index cases may be linked with an increased risk of developing type 1 diabetes in other siblings. Physicians need to be aware of this risk and screen the siblings if possible for better management. We have found some HLA loci associated with sib-pair antibody positive type 1 diabetes in our cohort of subjects from Qatar and believe more studies are required in the future to look for risk factors.

## Abbreviations

DKA: Diabetic Ketoacidosis
GAD65: Glutamic Acid Decarboxylase 65
IA-2A: Islet Antigen-2 Auto Antibody
IAA: Insulin Auto Antibody
MODY: Maturity Onset Diabetes of Young
ZnT8A: Zinc Transporter 8
